# Supplementary figures and images for: A skin colonizer disrupts inflammatory and humoral immune defenses in hidradenitis suppurativa
Source: EMBO Mol Med. 2026 Mar 24;18(5):1744–70. doi: 10.1038/s44321-026-00407-7 (PMC13179376; doi:10.1038/s44321-026-00407-7)

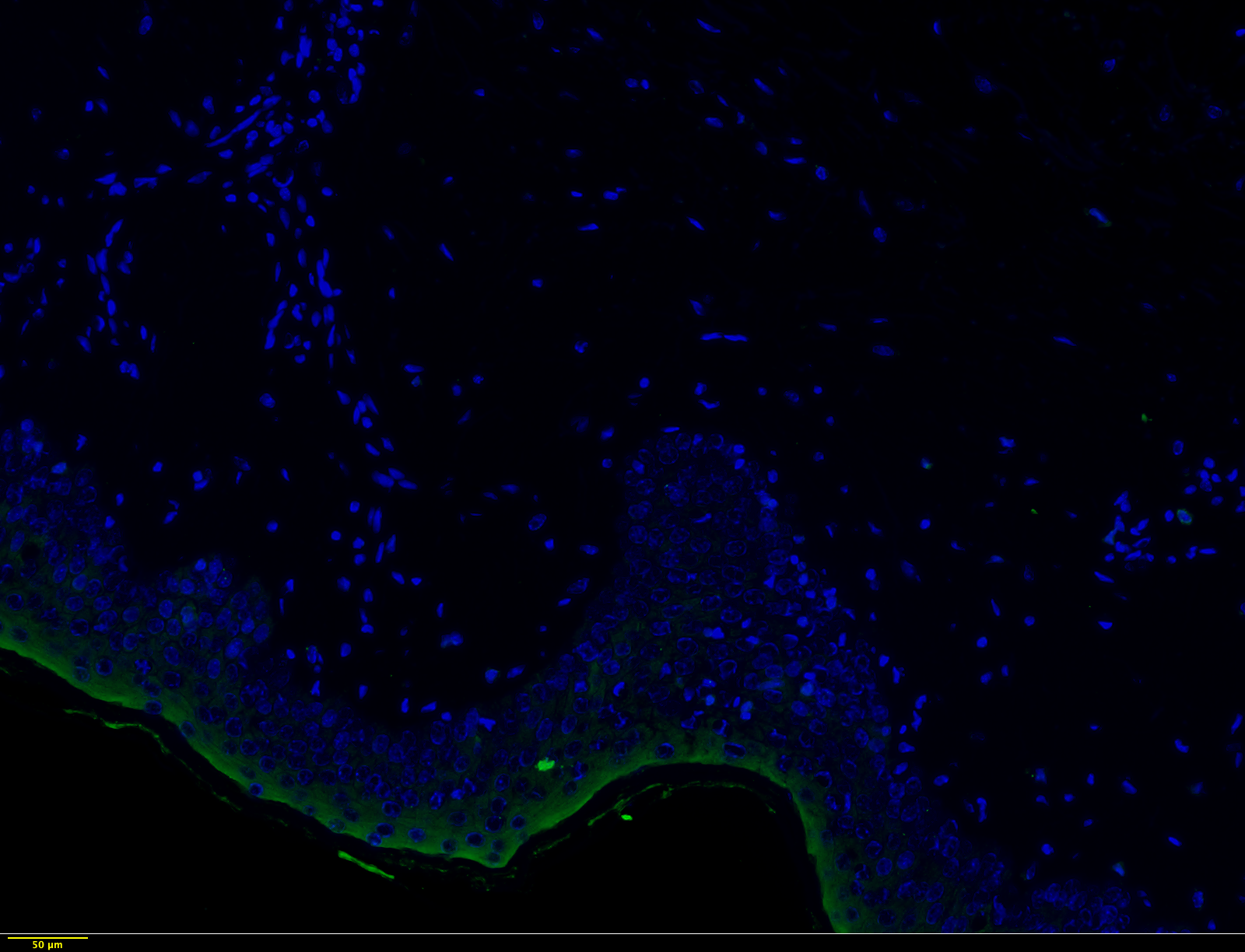

Supplement: Supplementary file 3 — Source data Fig. 2 [file 44321_2026_407_MOESM3_ESM.zip › Figure 2/2A/HS3 IgG.tif]

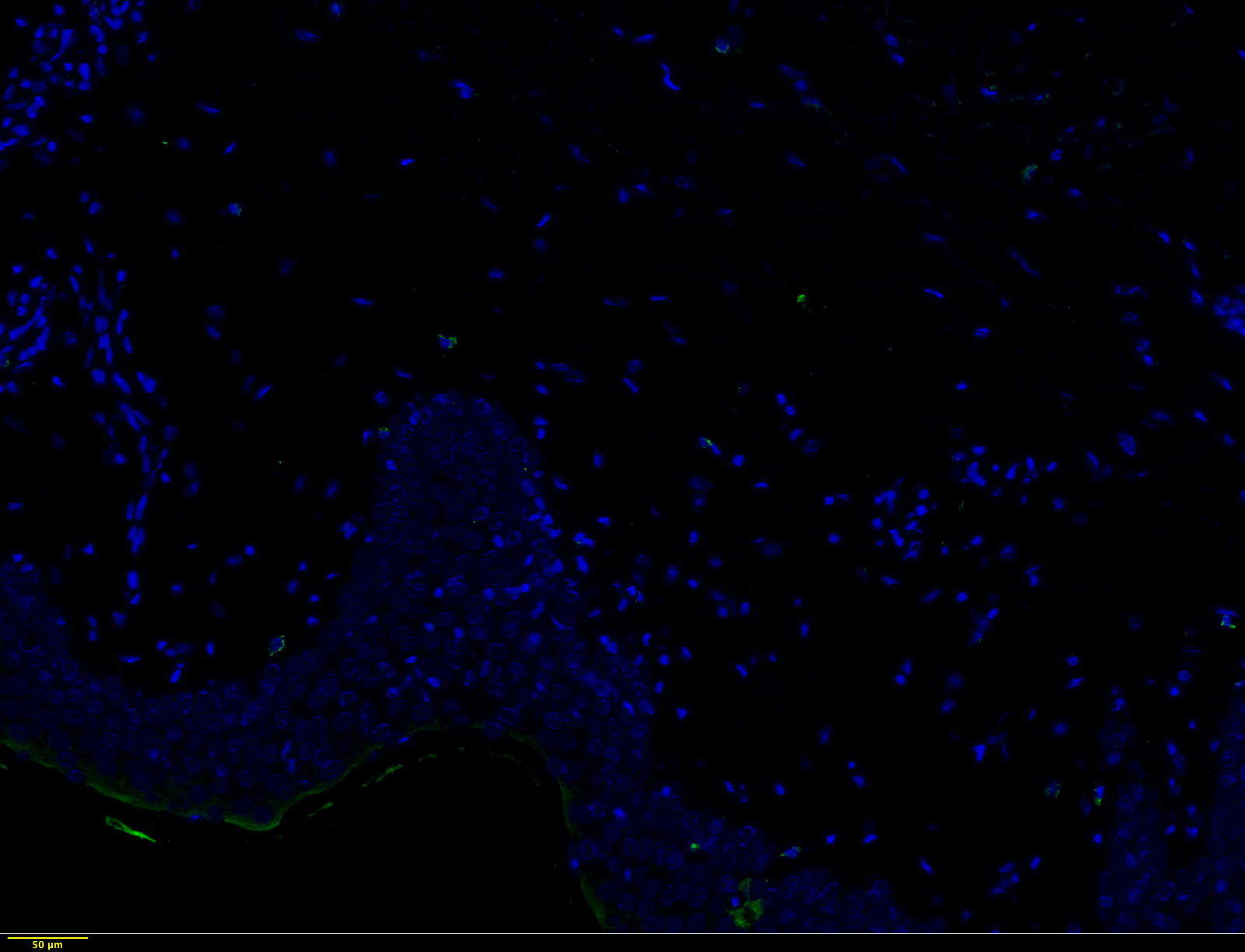

Supplement: Supplementary file 3 — Source data Fig. 2 [file 44321_2026_407_MOESM3_ESM.zip › Figure 2/2A/HC IgG.tif]

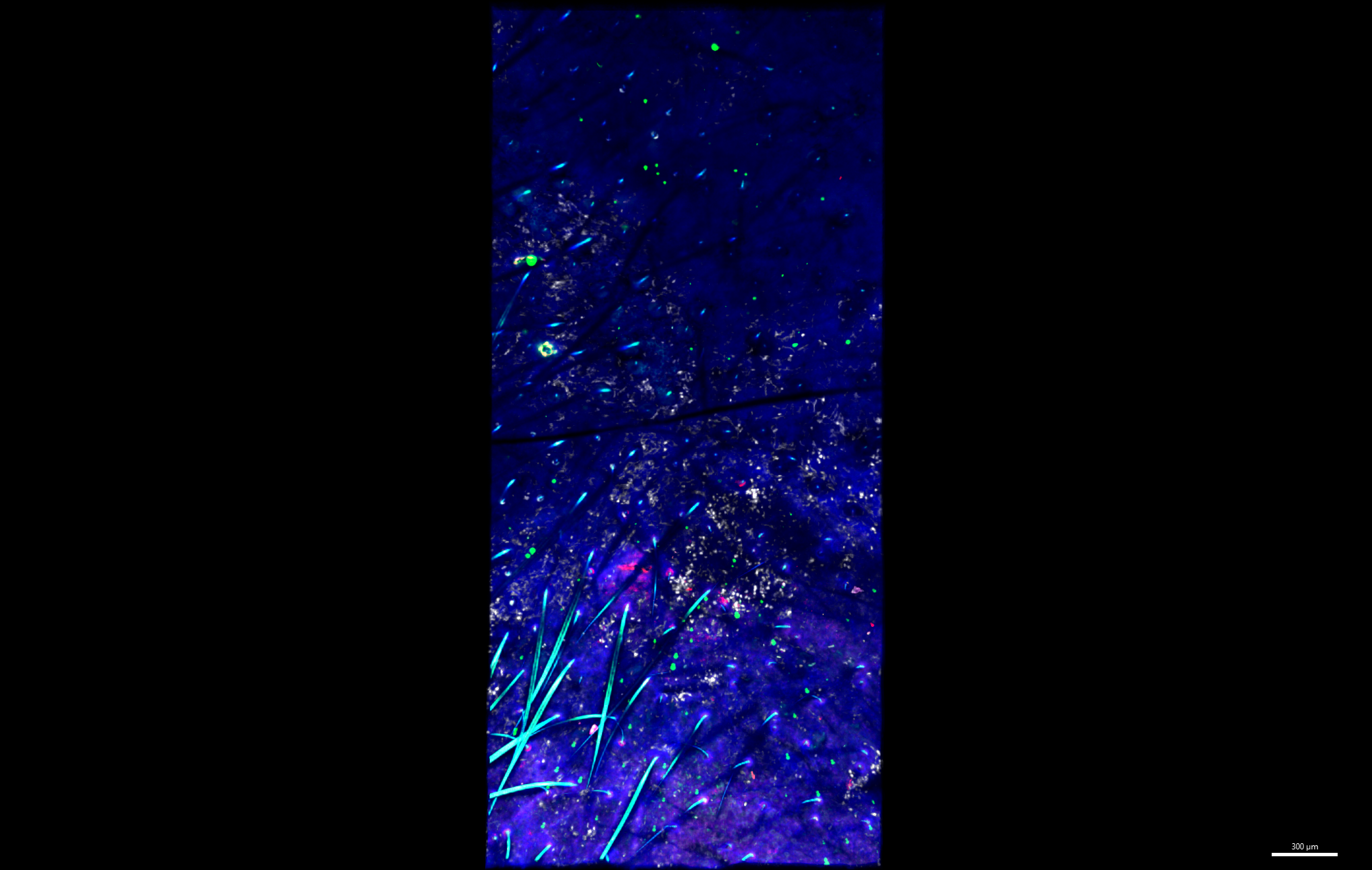

Supplement: Supplementary file 7 — Source data Fig. 6 [file 44321_2026_407_MOESM7_ESM.zip › Figure 6/6F/IgAFITC_CD138PE_CD4AF700_CD49fPB_CD19APC_e_CTRL_epidermis_I TileScan_001_Merging.tiff]

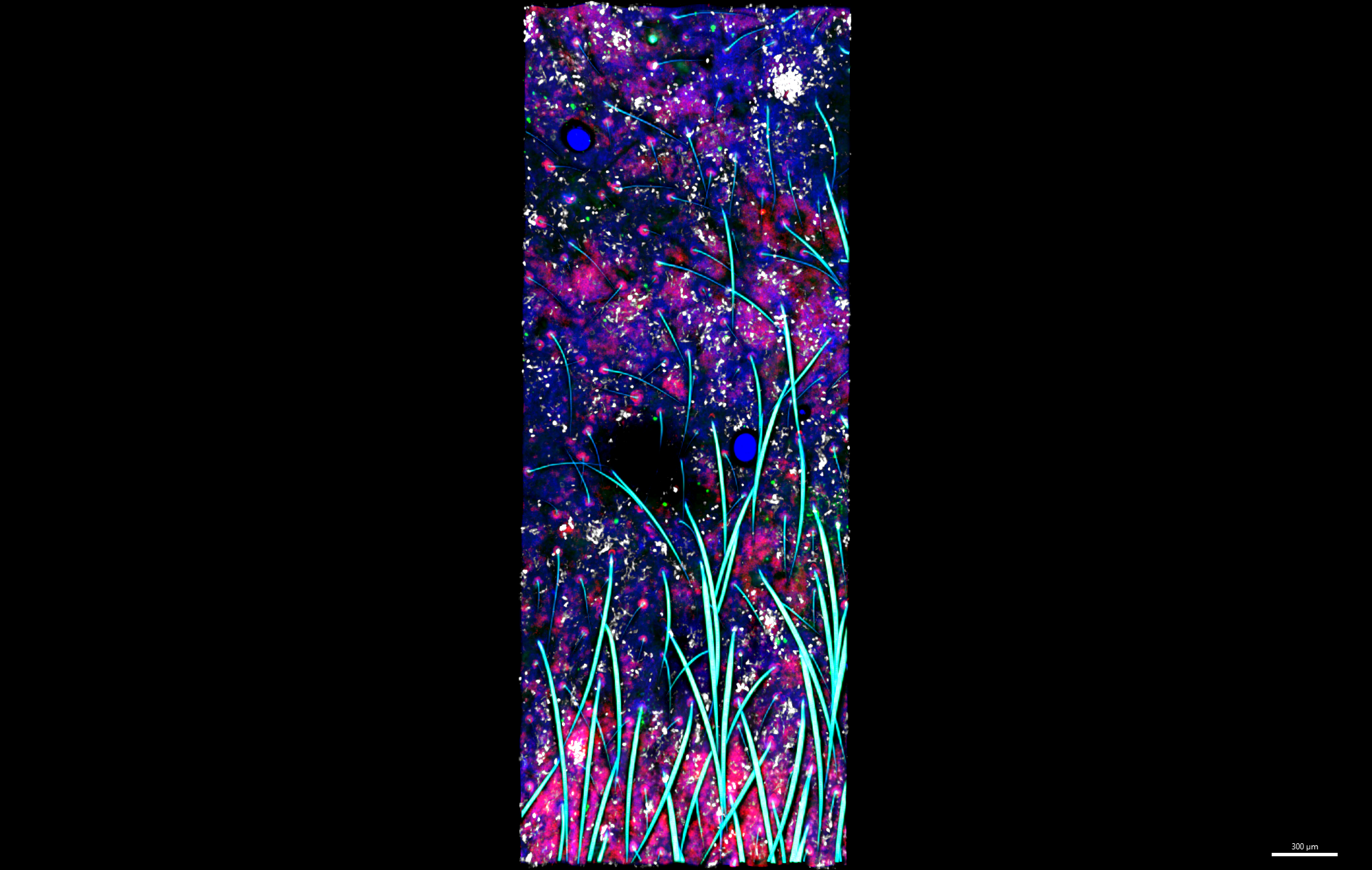

Supplement: Supplementary file 7 — Source data Fig. 6 [file 44321_2026_407_MOESM7_ESM.zip › Figure 6/6F/IgAFITC_CD138PE_CD4AF700_CD49fPB_CD19APC_d_PA_Epidermis_d60 TileScan_001_Merging.tiff]

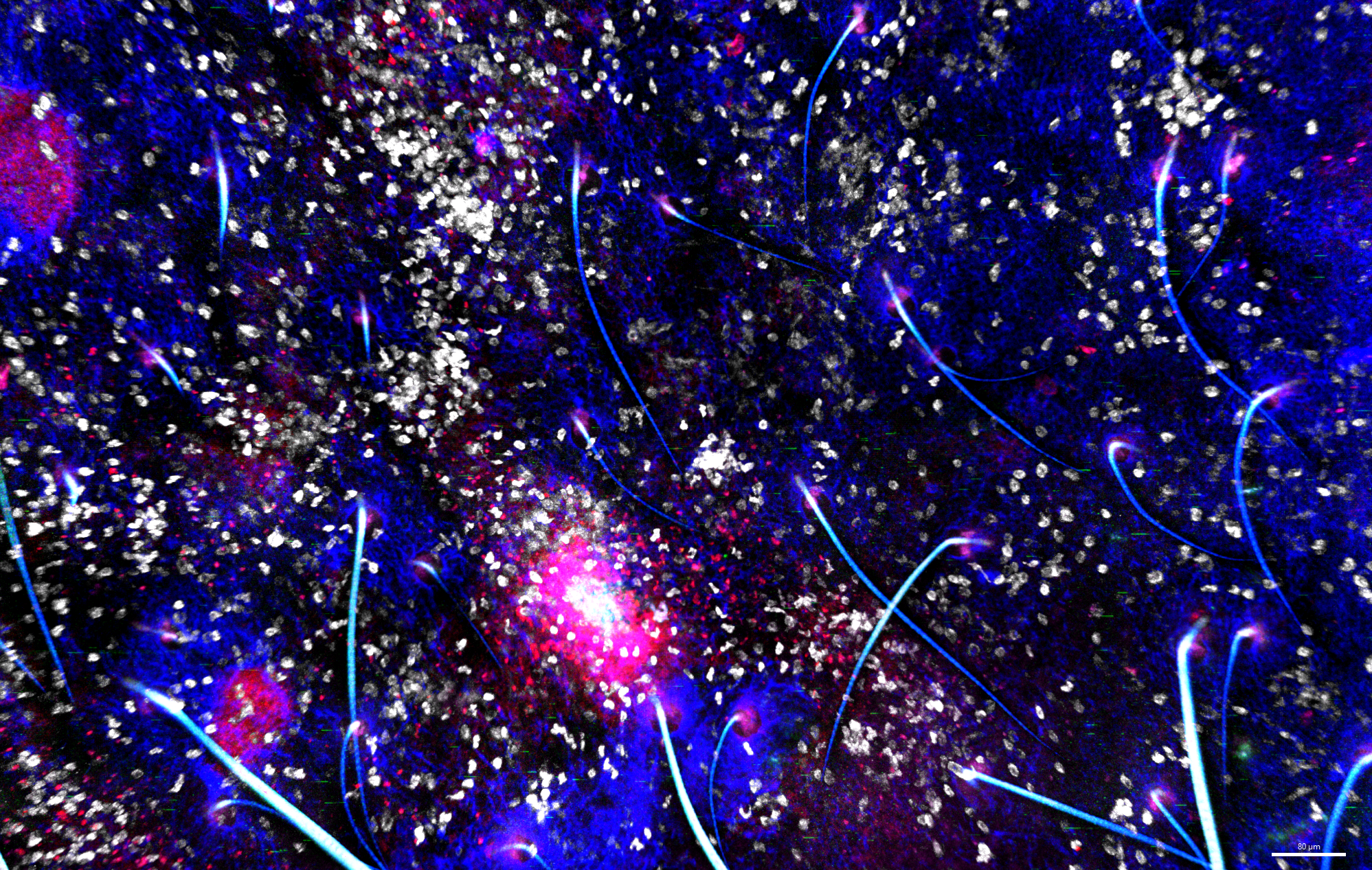

Supplement: Supplementary file 7 — Source data Fig. 6 [file 44321_2026_407_MOESM7_ESM.zip › Figure 6/6D/IgG2bFITC_CD138PE_CD19APC_CD49fPB_CD4AF700_a_PA_epidermis_TSB TileScan_001_Merging.tiff]

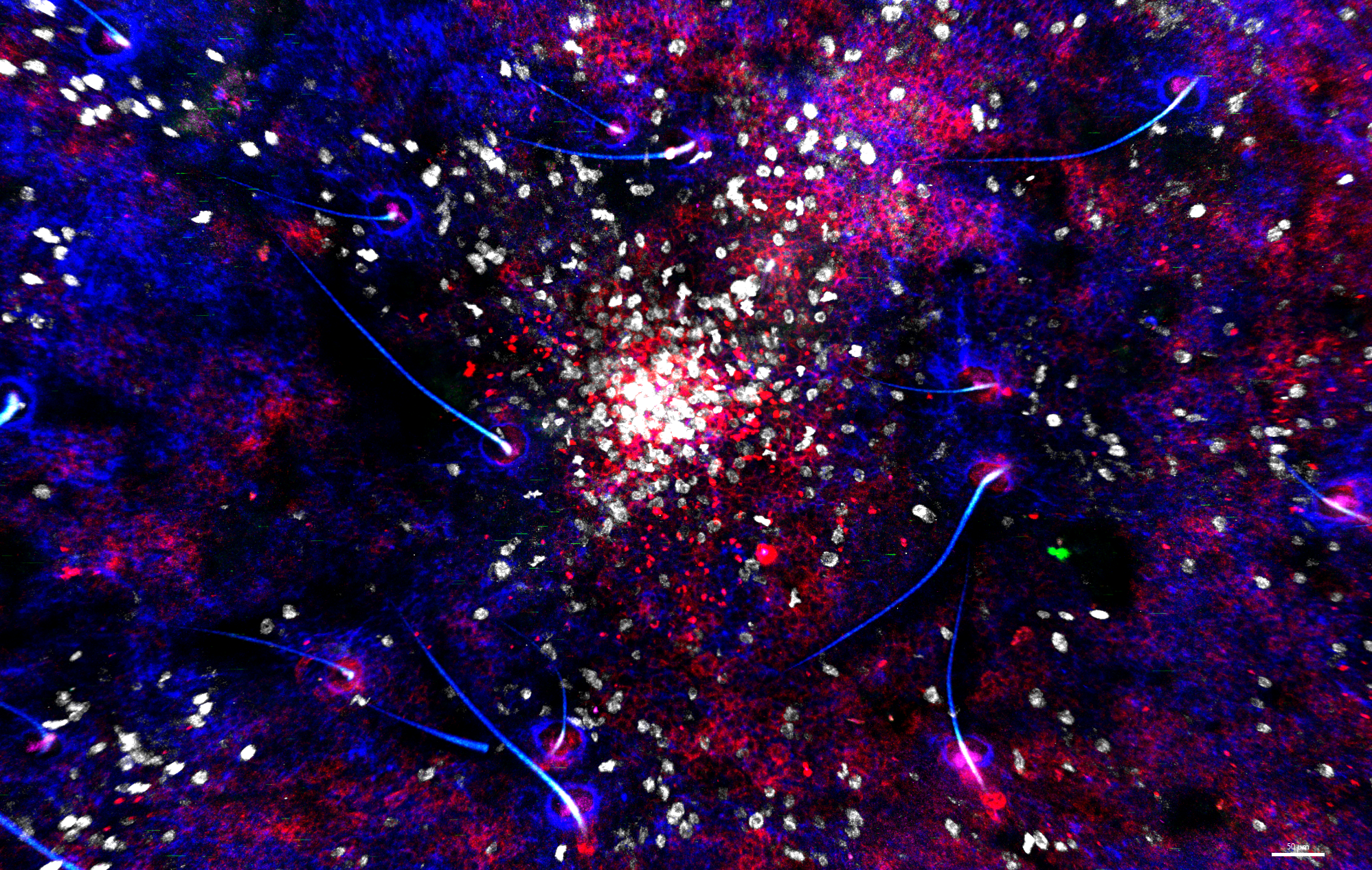

Supplement: Supplementary file 7 — Source data Fig. 6 [file 44321_2026_407_MOESM7_ESM.zip › Figure 6/6D/CD4AF700_CD49FPB_IgG2bFITC_CD138PE_CD19APC_c_Epi_d14_PA TileScan_001_Merging.tiff]
