# Supplementary material for: A skin colonizer disrupts inflammatory and humoral immune defenses in hidradenitis suppurativa
Source: EMBO Mol Med. 2026 Mar 24;18(5):1744–70. doi: 10.1038/s44321-026-00407-7 (PMC13179376; doi:10.1038/s44321-026-00407-7)
Supplement: Supplementary file 5 — Source data Fig. 4 [file 44321_2026_407_MOESM5_ESM.zip › Figure 4/read me.docx]

Légende Couleur

Blue> Dapi chanel 2

Red> IgA (AF488) chanel 0

Green> IgG (AF647) chanel 3

Orange>CD138 (AF555) chanel 1
