# Supplementary material for: A skin colonizer disrupts inflammatory and humoral immune defenses in hidradenitis suppurativa
Source: EMBO Mol Med. 2026 Mar 24;18(5):1744–70. doi: 10.1038/s44321-026-00407-7 (PMC13179376; doi:10.1038/s44321-026-00407-7)
Supplement: Supplementary file 8 — Expanded View Figures [file 44321_2026_407_MOESM8_ESM.pdf]

Expanded View Figures

Figure EV1. Anti-*P. disiens* and *S. lugdunensis* Ig responses and sex effect on anti-bacterial Ig responses.

(**A**) Relative level of serum IgA/Gs binding to *P. disiens* and *S. lugdunensis* in HS1 ($n = 29$), HS2 ($n = 17$), HS3 ($n = 11$), or AD patients ($n = 7$), compared to HC ($n = 32$). (**B**) Heatmap comparing mean levels of anti-bacterial IgAs, IgGs, and IgMs in sera of male (M) or female (F) HS1/2/3 patients to those of HC (left), to each other (middle) or to those of AD patients (right). Statistical analyses used a moderated, FDR-corrected two-tailed t-test (limma). *$p < 0.05$, **$p < 0.01$, ***$p < 0.001$, and ****$p < 0.0001$.

© The Author(s)

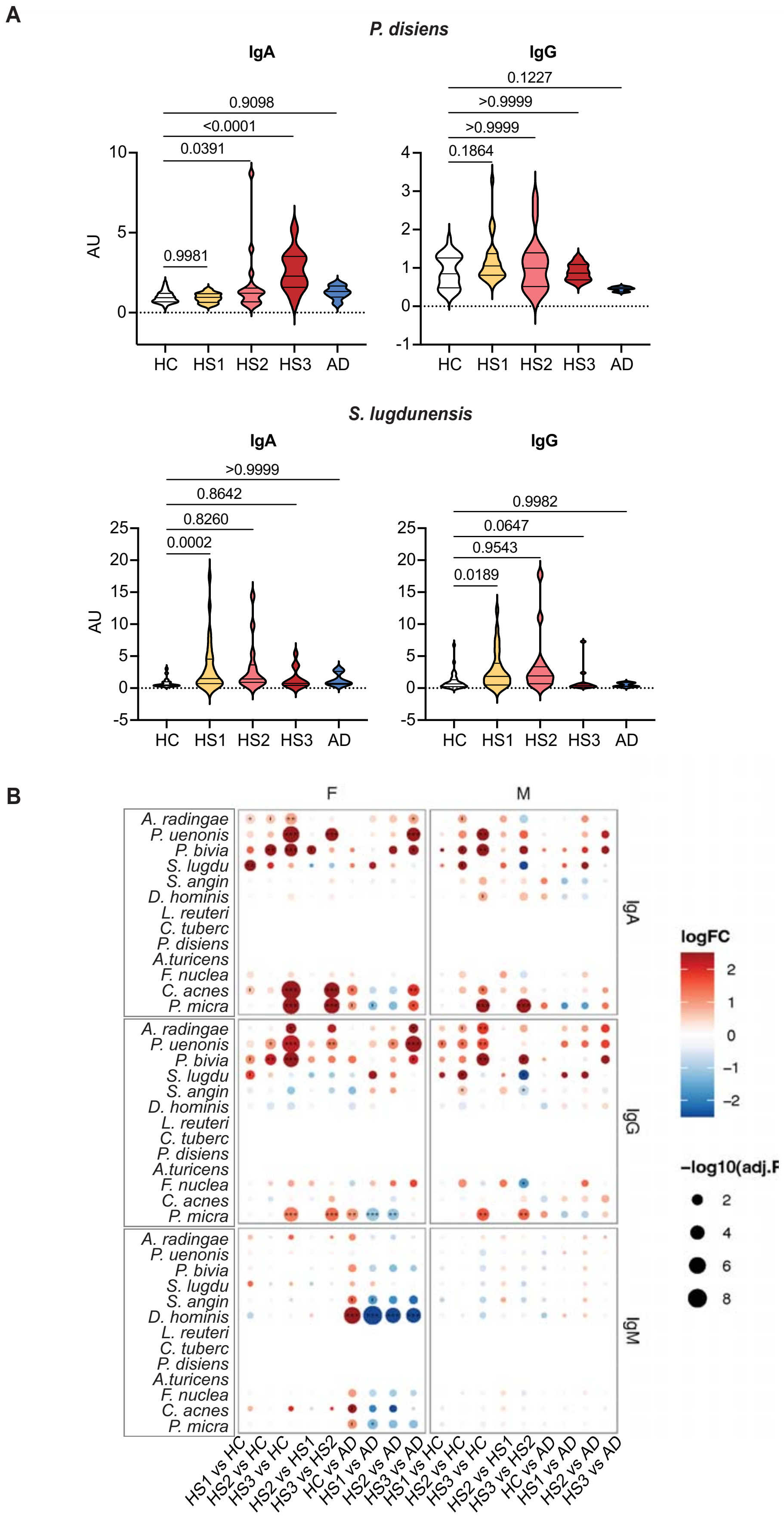
A
P. disiens
IgA
IgG
0.9098
<0.0001
0.0391
0.9981
0.1227
>0.9999
>0.9999
0.1864
AU
HC HS1 HS2 HS3 AD
S. lugdunensis
>0.9999
0.8642
0.8260
0.0002
0.9982
0.0647
0.9543
0.0189
B
F
M
A. radingae
P. uenonis
P. bivia
S. lugdu
S. angin
D. hominis
L. reuteri
C. tuberc
P. disiens
A.turicens
F. nuclea
C. acnes
P. micra
IgA
IgG
IgM
logFC
−log10(adj.P
HS1 vs HC
HS2 vs HC
HS3 vs HC
HS2 vs HS1
HS3 vs HS2
HC vs AD
HS1 vs AD
HS2 vs AD
HS3 vs AD

 © The Author(s)

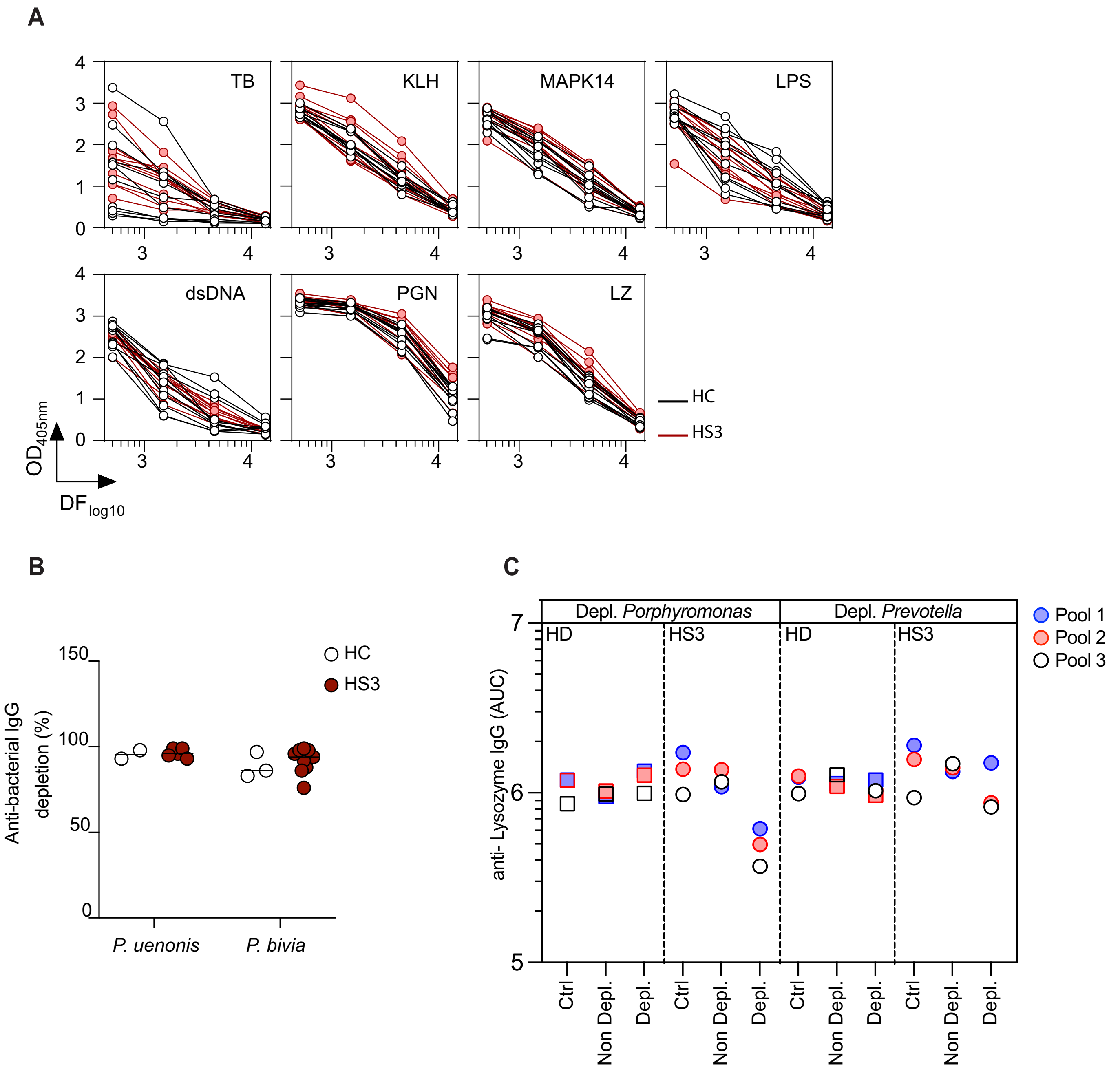

Figure EV2. IgG autoreactivity and efficacy of anti-bacterial IgG depletion.

(**A**) ELISA titration curves of individual HC (black) and HS3 (red) sera for each antigen tested. (**B**) Loss of anti-bacterial IgG (%), as measured by flow cytometry, in independent pools of HC ($n = 2, 3$) and HS3 sera ($n = 5, 9$) sera following depletion on *P. uenonis* or *P. bivia*, respectively. (**C**) ELISA titration curves of pooled HC (black) and HS3 (red) sera against LZ following depletion on *P. uenonis* or *P. bivia*.

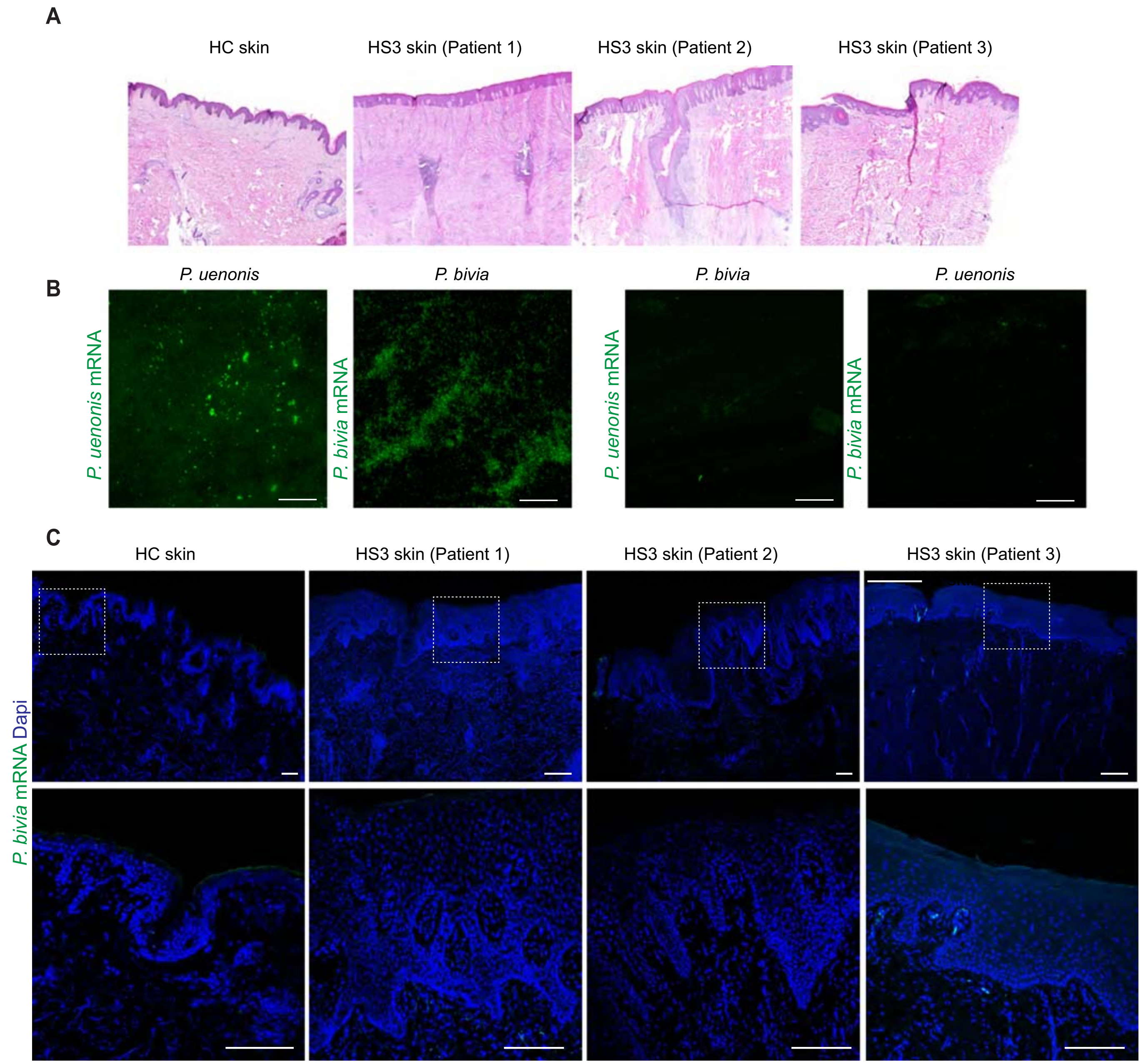

Figure EV3. ***Prevotella bivia*** **does not invade the epidermis in severe HS.**

(**A**) Representative images of skin samples from HC and 3 HS3 patients stained by H&E. (**B**) Validation of mRNA probes targeting *P. uenonis* and *P. bivia* on their respective live bacterial culture (left). Each probe was also tested on the other species (right) as a negative control to assess specificity. (**C**) Representative images of skin samples from HC and 3 HS3 patients hybridized with anti-*P. bivia* mRNA probes, with nuclei counterstained with Dapi. Dotted square indicates the magnified area (bottom panel, Scale bars: 500 μm).

 © The Author(s)

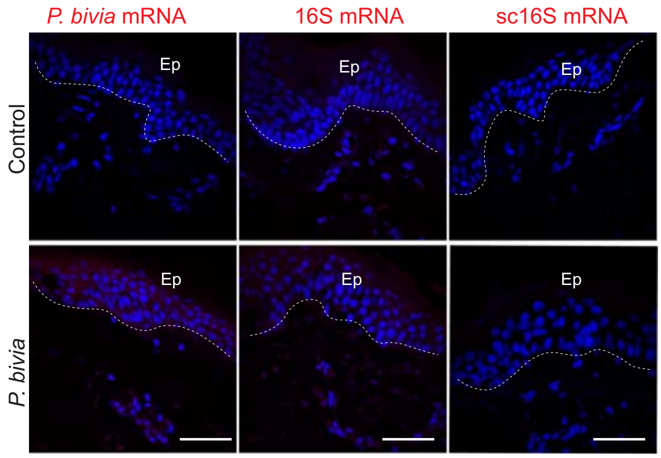

Figure EV4. Lack of bacterial invasion in *P. bivia*-coated skin explants.

Representative images of skin explant sections hybridized with an anti-*P. bivia* mRNA probe, pan-bacteria 16S mRNA, or scrambled 16S mRNA probes as controls. Nuclei were stained in DAPI (blue). Scale bars: 50 µm.

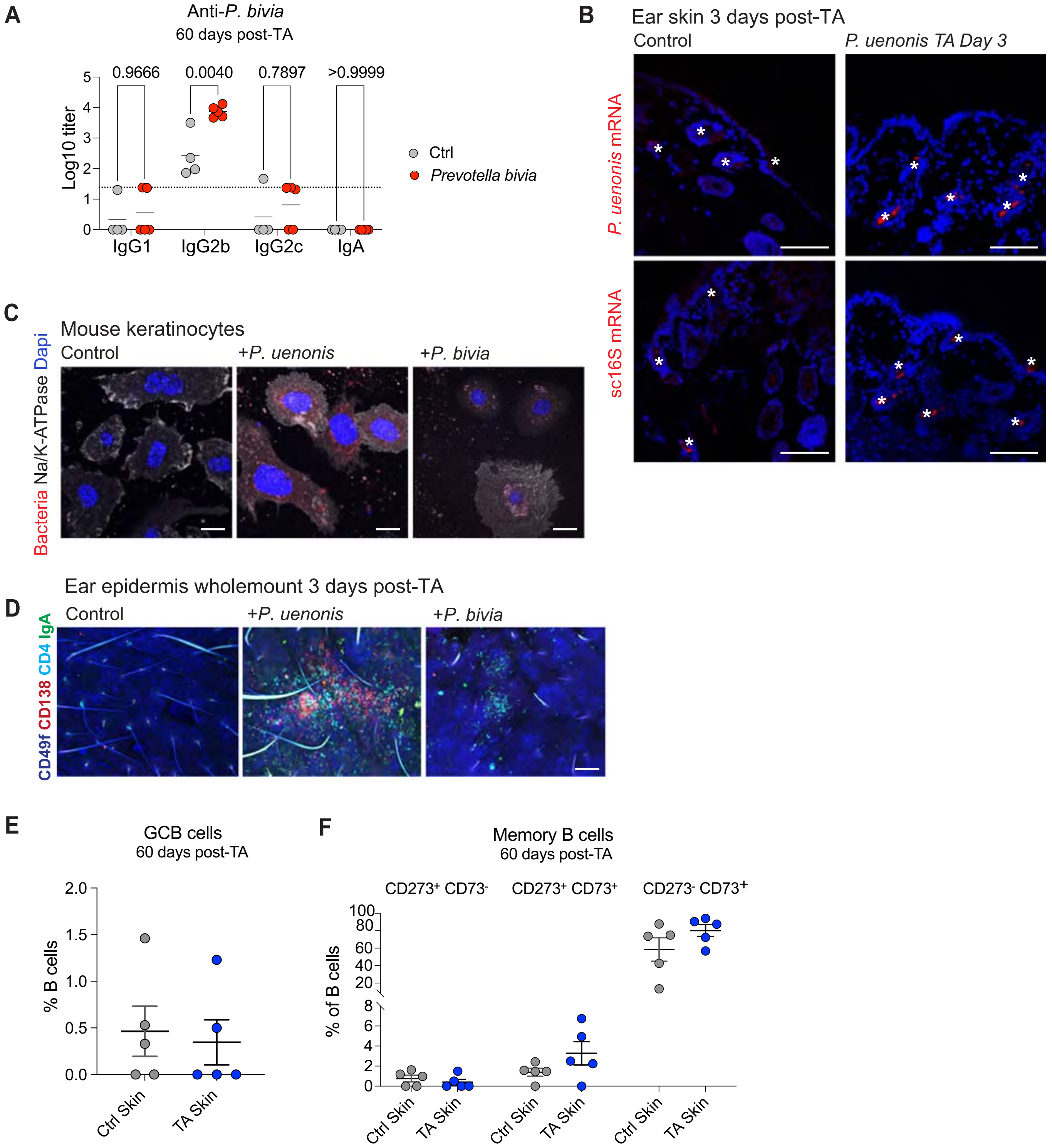
A
Anti-P. bivia
60 days post-TA
0.9666
0.0040
0.7897
>0.9999
Log10 titer
IgG1
IgG2b
IgG2c
IgA
Ctrl
Prevotella bivia
B
Ear skin 3 days post-TA
Control
P. uenonis TA Day 3
P. uenonis mRNA
sc16S mRNA
C
Mouse keratinocytes
Control
+P. uenonis
+P. bivia
Bacteria Na/K-ATPase Dapi
D
Ear epidermis wholemount 3 days post-TA
Control
+P. uenonis
+P. bivia
CD49f CD138 CD4 IgA
E
GCB cells
60 days post-TA
% B cells
Ctrl Skin
TA Skin
F
Memory B cells
60 days post-TA
CD273+ CD73-
CD273+ CD73+
CD273- CD73+
% of B cells
Ctrl Skin
TA Skin

 © The Author(s)

Figure EV5. Topical association with *P. uenonis* triggers PC recruitment in vivo.

(**A**) Serum levels of anti-P. *Bivia* IgG1, IgG2b, IgG2c, and IgA were measured in control and TA groups at 60-days post-TA. Each dot represents an individual mouse. Data were mean Log10 Ig titers in each individual mouse ($n = 5$) ± SD. Statistical significance was assessed using two-way ANOVA with Bonferroni's multiple comparisons test. (**B**) Confocal images of ear skin sections from control and *P. uenonis*-associated mice 3 days post-TA, hybridized with an anti-*P. uenonis* mRNA or scrambled 16S mRNA probe as control (red), with nuclei counterstained with DAPI (blue). White stars indicate non-specific labeling of hair follicles. Scale bars: 100 µm. (**C**) KCs isolated from wild-type mice were exposed to fluorescently labeled *P. uenonis*, *P. bivia*, or broth (Ctrl) for 48 h under anaerobic conditions. Confocal images show KCs infected with bacteria, Dapi-stained nuclei, and membrane marker Na, K-ATPase. Scale bar: 10 µm. (**D**) Representative confocal images of mouse ears 3 days post-TA. Control (left), *P. uenonis*-associated (middle), and *P. bivia*-associated (right) mice are shown. Ears were stained for CD49f (KCs), CD138 (PCs), CD4 (helper T cells) and IgA-expressing cells. Scale bars: 80 µm. (**E**) FACS analysis of germinal center B cells (GCBs) in ear skin 60 days post-TA with *P. uenonis*. Data were mean % in each individual mouse ($n = 5$) ± SD. (**F**) FACS analysis of memory B cell subsets (defined by their expression of CD273 and/or CD73 markers) in ear skin 60 days post-TA with *P. uenonis*. Data were mean % in each individual mouse ($n = 5$) ± SD.
